# Case of Glioblastoma Multiforme in the Left Temporoparietal Region of the Brain

**DOI:** 10.7759/cureus.28621

**Published:** 2022-08-31

**Authors:** Rounak Chandnani, Ashish Anjankar

**Affiliations:** 1 Medicine and Surgery, Jawaharlal Nehru Medical College, Wardha, IND; 2 Biochemistry, Jawaharlal Nehru Medical College, Wardha, IND

**Keywords:** phosphoinositide 3-kinase (pi3k), rat sarcoma (ras), pet scans (positron emission tomography), receptor tyrosine kinase (rtk), retinoblastoma protein (rb)

## Abstract

Glioblastoma is one of the most frequent and malignant primary brain or spinal cord cancers. We present the case of a 48-year-old man diagnosed with a grade IV histology tumor, which is the most fatal according to WHO classification. Mutations in the p53, retinoblastoma protein (RB), receptor tyrosine kinase (RTK), rat sarcoma (RAS), and phosphoinositide 3-kinase (PI3K) signaling genes are frequently seen in glioblastoma. Radiation therapy, alkylating chemotherapy, and surgery are often used as glioblastoma treatments. O6-methylguanyl DNA methyltransferase (MGMT) promoter methylation predicts the effectiveness of alkylating chemotherapy with temozolomide, which directs the selection of first-line therapy in elderly patients. Glioblastoma goes unnoticed because of age-related factors, yet it is recognized that older people are more prone to getting it. The patient also had nausea, vomiting, and headaches. The disease's course was slowed while the patient's signs and symptoms were lessened by the treatment. The doctor checks reflexes, eyesight, hearing, coordination, and more. MRI is the most reliable tool for locating glial tumours. It is also possible to do additional diagnostic procedures like CT or positron emission tomography (PET) scans. A biopsy may also be carried out, depending on the circumstances and the location of the tumor. A biopsy's objective is to identify the cell's kind and the amount of its dissemination; specialized tests carried out by medical professionals and technicians reveal the prognosis and better treatment alternatives. The only treatments accessible now are surgery, chemotherapy, radiation, tumor treating fields therapy, targeted medication therapy, and palliative care. It is anticipated that there will be fewer fatalities in the future.

## Introduction

The majority of glioblastoma patients receive radiochemotherapy and surgical tumor excision as conventional treatments. But eventually, nearly every tumor returns. The majority of the time, recurrent cancers have already penetrated functioning brain regions, making them less responsive to therapy than the original tumor and prohibiting further surgical excision. Recurrent glioblastoma currently has no conventional treatment, and patients often pass away from these tumors 12 to 15 months after first diagnosis [1]. The primary brain tumor that strikes adults the most frequently and fatally is glioblastoma. Grade IV, the highest grade in the World Health Organization (WHO) classification of brain tumors, is characterized histopathologically by necrosis and endothelial growth. According to conventional clinical terminology, "secondary glioblastoma" is used to describe the minority of glioblastomas that arise from previously diagnosed WHO grade II or grade III gliomas. Most glioblastomas are isocitrate dehydrogenase (IDH) wild-type; however, some point mutations of the genes encoding isocitrate dehydrogenase 1 or 2 appear to molecularly distinguish certain tumors that are connected with younger age and a better prognosis [2].

Patients with a primary brain tumor are likely to be seen for the first time by interns, private doctors, and emergency room doctors, who generally continue to be engaged in their care throughout the entire course of the disease. Malignant tumors have a poor prognosis and immediately impact a person's quality of life and cognitive ability [3]. Primary brain tumors have been linked to a number of known risk factors, including therapeutic ionizing radiation exposure, labor in the production or refining of petroleum, exposure to vinyl chloride or pesticides, and employment in the synthetic rubber industry. Ionizing radiation therapy poses a significant risk of developing brain tumors. Patients with glioblastoma had a high prevalence (17%) of past therapeutic irradiation. Multiple studies noted an elevated risk of brain tumors in those who had irradiation for leukemia as children [4]. The parallel identification of cell populations with varying degrees of differentiation and cellular and morphological heterogeneity are important histological characteristics of glioblastomas. Furthermore, glioblastomas have a wide range of genetic anomalies. Because distinct cell types inside the tumor tissue may react to medicines differently, this tumor heterogeneity may make it difficult to treat the condition. Identifying and characterizing distinctive subpopulations responsible for particular disease pathologies is one method for solving these issues [5].

The standard of care for most patients with newly diagnosed glioblastoma consists of surgery as soon as it is safe to do so, followed by extensive field radiation, in addition to concurrent and up to six maintenance cycles of temozolomide chemotherapy. No other treatment intervention that treats tumors has been demonstrated to increase overall survival in newly diagnosed patients. The care guidelines for recurrence are less clear for all glioblastomas, which eventually advance. A tiny percentage of patients who experience localized recurrence are given a second surgery or re-irradiation, although none of these procedures has been proven to increase survival time. Most clinical trial strategies targeting intrinsic glioblastoma targets take into account tyrosine receptor kinase-mediated oncogenic signaling, cell cycle regulation, and vulnerability to apoptosis induction [6].

## Case presentation

**Figure 1:**
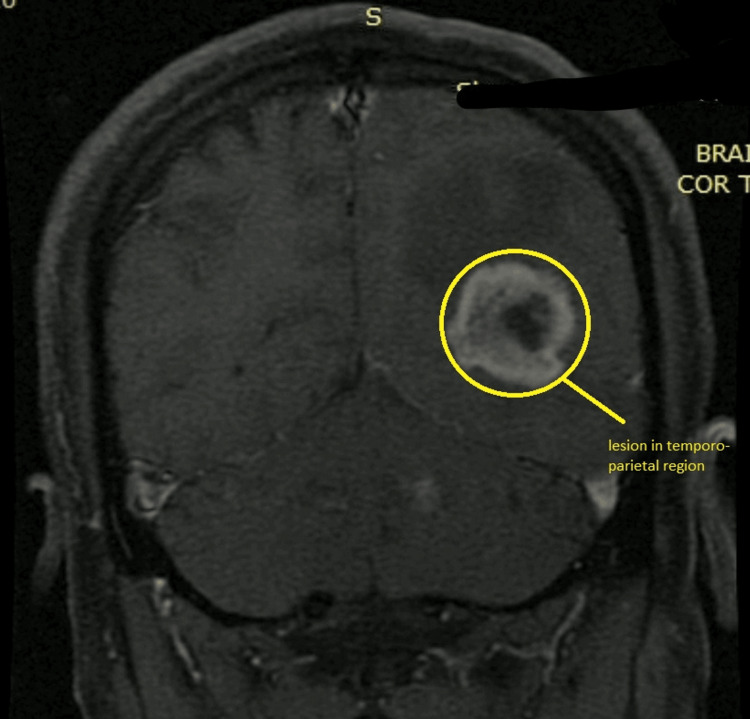
Coronal view of the MRI showing lesion in the left temporoparietal region

**Figure 2:**
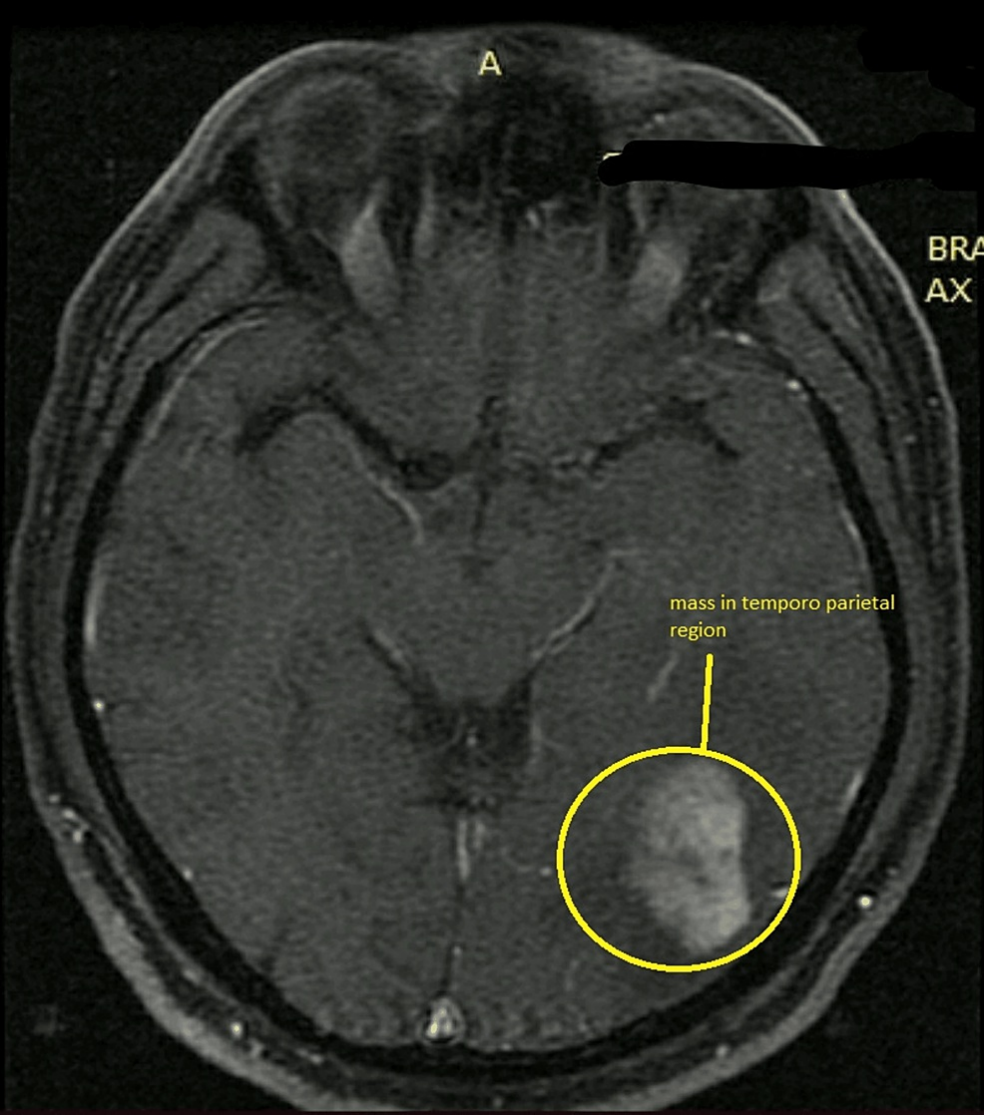
Axial view of the MRI showing lesion in the left temporoparietal region

**Figure 3:**
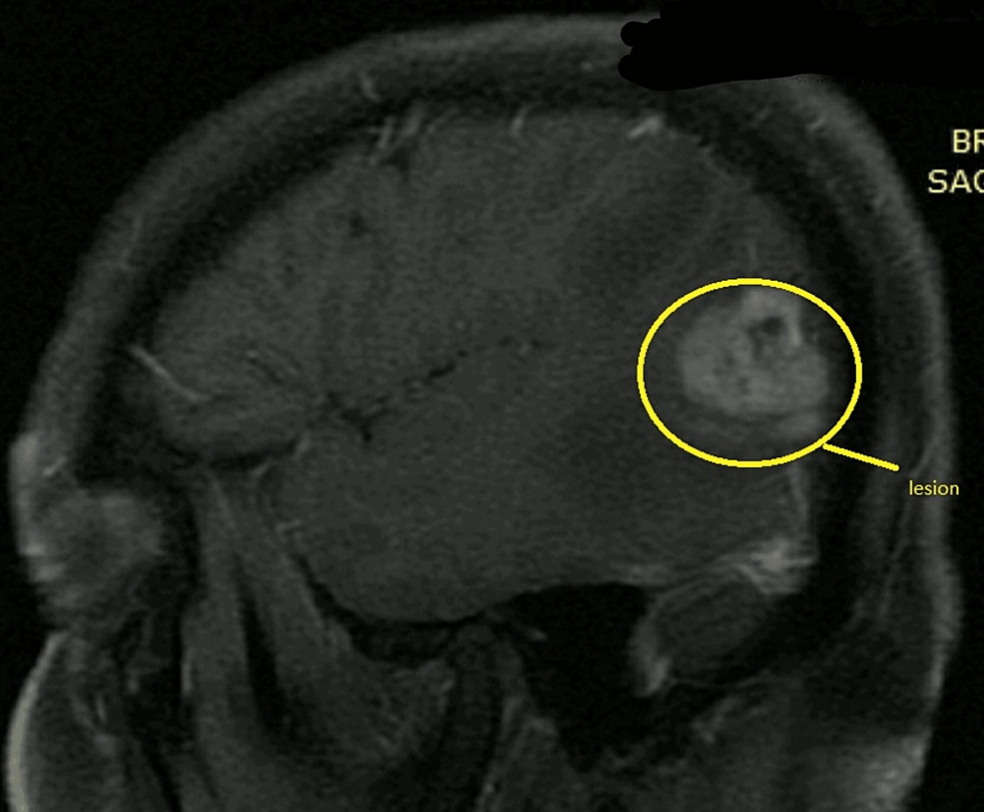
Sagittal view of the MRI showing lesion in the left temporoparietal region

A 48-year-old patient came to the department with complaints of headache that was insidious in onset and moderate to severe in nature, and unable to concentrate and focus on work. The patient had no history of migraine, meningitis, diabetes, blood pressure, or acute transient ischemic attacks. The patient was suffering from headaches seven days before diagnosis; before that, the patient was typical with no history of headaches. Hence MRI was suggested to identify the cause of the symptoms. Regional sulci were narrowed with a slight hyperintensity in the left medial temporal area. Grey matter differentiation was well maintained in all aspects, and cortical sulci, basal cisterns, and Sylvian fissures were affected. The entire ventricular system was well visualized and narrowed. Sellar and parasellar structures were normal. The pituitary gland was deformed. Vascular structures at the base of the skull and dural venous sinus showed normal flow void. The cerebellum and brainstem did not reveal any focal lesion. Findings indicated malignant neoplastic mass in the left posterior temporoparietal region with severe perifocal edema-hyperintensity in the left medial temporal lobe. Figures 1-3 show the lesion's location in axial, coronal, and sagittal views with multiplanar, multi-echo plain, and contrast brain MRI done on the 1.5 Tesla system, revealing a large brightly heterogeneously enhancing mass in the left posterior temporal region showing severe perifocal edema.

Figures showed a large bright but heterogeneous enhancing mass in the left posterior temporoparietal region measuring about 45x31x33 mm with severe perifocal edema. After the MRI, the patient was referred to a super-specialty hospital for further treatment, where a biopsy was performed, and the patient received six stitches. Tissue from the lesion in the left temporoparietal region was taken. Regional sulci were narrowed with slight hyperintensity in the left medial temporal region, suggestive of high-grade glioma. Frozen section diagnosis suggested tissue from the left temporoparietal area with high-grade glioma. Gross direct specimen examination showed tissue from the left temporoparietal region. The patient received multiple whitish tissue bits aggregating to 1x0.8x0.3 mm. Two tissue bits were submitted for frozen study: Fro-A and Fro-B. One was used for squash smear examination, while the remaining tissue was processed for paraffin study. After microscopic examination, sections showed a highly cellular tumor composed of atypical glial cells, many multinucleated cells, and giant cells. Nuclear hyperchromasia, pleomorphism, and acute mitotic activity were noted with the presence of prominent microvascular proliferation and palisade foci.** **While tumor cells are harmful to isocitrate dehydrogenase (IDH1R132), they diffusely display glial fibrillary acidic protein (GFAP) and p53 on immunohistochemistry performed with acceptable control. In hotspot locations, the Ki67 antigen proliferation index is 40%.** **After removing stitches, the patient was suggested six cycles of radiation. After six cycles, a 45-day gap and then three cycles of oral radiation were asked to be performed. The patient performed three cycles of radiation therapy and was admitted for further treatment in the hospital.

## Discussion

Even if the patient responds well to the treatment, soluble urokinase-type plasminogen activator receptor biomarkers seem to be particularly useful in association with other biomarkers for a modest mean value of 15 to 18 months.** **In fibrinolytic pathways, the nonsoluble version of suPAR known as urokinase plasminogen activator receptor (or CD87) is expressed on immune cells such as neutrophils, monocytes, macrophages, and lymphocytes [7]. Risk factors for glioblastoma development are poorly defined; males are affected more commonly than females, and it has no apparent cause [2]. The most frequent and aggressive brain tumor is glioblastoma. The majority of glioblastomas that develop in older patients start from scratch without any histological or clinical signs of a less serious precursor illness. Anaplastic or diffuse low-grade astrocytomas develop into secondary glioblastomas. They manifest in younger people, have less necrosis, are more often seen in the frontal lobe, and have a noticeably better prognosis. Despite being nearly similar histologically, the genetic and epigenetic profiles of primary and secondary glioblastomas vary. Significant genetic markers for this malignancy are isocitrate dehydrogenase 1 (IDH1)mutations, which are connected to a hypermethylation phenotype and are seen in secondary glioblastomas but not prominent glioblastomas. IDH1 mutations are the first genetic changes to be discovered in precursor low-grade diffuse astrocytomas and oligodendrogliomas, showing that these tumors are derived from neural precursor cells that are different from those of primary glioblastomas. Here, we cover the biological consequences of IDH1 mutations and the epidemiological, clinical, histological, genetic, and expression features of primary and secondary glioblastomas. In light of this, we draw the conclusion that this genetic mutation is a clear molecular marker of secondary glioblastomas and is more precise and objective than clinical criteria [8].

Investigations indicated that IDH mutation status, gender, age, and postoperative adjuvant therapy were all independent indicators of prognosis for glioblastoma patients, supporting prior findings. In other words, individuals who had postoperative chemoradiotherapy had better prognoses. O^6^-methylguanyl DNA methyltransferase (MGMT) methylation is a supportive element for patient prognosis since it serves as the main prognostic predictor for response to alkylating agent therapy. It was shown that safe resection should be maximized, which confirmed the accepted wisdom for treating glioblastoma. One crucial aspect affecting the patient's prognosis is the type of postoperative chemotherapy used. But while the IDH1 missing rate significantly decreases, the percentage of glioblastoma patients undergoing postoperative chemoradiotherapy increases. One of the most critical aspects of glioma diagnosis and treatment nowadays is molecular pathology. A commonly known misconception concerning glioblastoma is that the sole treatment option for this fatal condition is surgical resection. We reasoned that, in recent years, neurosurgical multimodal technologies have made it possible to sufficiently remove each lesion, leading to safer tumor removal and the establishment of a new standard of care for the removal of glioblastomas. One of the predictive and prognostic markers for improved outcomes in glioblastoma patients has previously been recognized as using tools like intra-operative mapping and MRI. Up to 50% of original malignant tumors of the central nervous system (CNS) are glioblastomas. Glioblastomas' invasive and aggressive character makes them virtually always deadly, even with the best surgical and postoperative adjuvant therapy [9].

## Conclusions

Even though malignant glioma is still an incurable condition, there are now more and better treatment options available thanks to increased knowledge of the intricate molecular biology of these tumors, their microenvironment, and immunologic interactions with the host. Trials using targeted drugs that target receptor tyrosine kinases and signal transduction pathways are among the novel potential treatments being considered. We are gradually accepting a more dynamic picture of glioblastomas instead of seeing them as uniform, static tumors with strict hereditary features.
